# Targeting galectin-3 with a high-affinity antibody for inhibition of high-grade serous ovarian cancer and other MUC16/CA-125-expressing malignancies

**DOI:** 10.1038/s41598-021-82686-3

**Published:** 2021-02-12

**Authors:** Marina Stasenko, Evan Smith, Oladapo Yeku, Kay J. Park, Ian Laster, Kwangkook Lee, Sven Walderich, Elizabeth Spriggs, Bo Rueda, Britta Weigelt, Dmitriy Zamarin, Thapi Dharma Rao, David R. Spriggs

**Affiliations:** 1 Gynecology Service, Department of Surgery, Memorial Sloan Kettering Cancer Center, New York, NY 10065 USA; 2 Division of Gynecologic Oncology, Department of Obstetrics and Gynecology, NYU Langone Health, New York, NY 10016 USA; 3 Department of Medical Oncology, Monoclonal Antibody Core, Dana-Farber Cancer Institute, Boston, MA 02215 USA; 4 Division of Hematology-Oncology, Massachusetts General Hospital, 55 Fruit St, Boston, MA 02114 USA; 5 Department of Medicine, Massachusetts General Hospital, Boston, MA 02114 USA; 6 Department of Pathology, Memorial Sloan Kettering Cancer Center, New York, NY 10065 USA; 7 Department of Medicine, University of California San Francisco Medical Center, San Francisco, CA 94143 USA; 8 Arnold Arboretum of Harvard University, Boston, MA 02131 USA; 9 Department of Obstetrics and Gynecology, Vincent Center for Reproductive Biology, Massachusetts General Hospital, Boston, MA 02114 USA; 10 Department of Obstetrics, Gynecology, Reproductive Biology, Harvard Medical School, Boston, MA 02114 USA; 11 Gynecologic Medical Oncology Service, Department of Medicine, Memorial Sloan Kettering Cancer Center, New York, NY 10065 USA; 12 Department of Medicine, Weill Cornell Medical College, New York, NY 10065 USA

**Keywords:** Ovarian cancer, Cancer microenvironment, Cancer therapy, Metastasis, Cancer, Immunology

## Abstract

The lectin, galectin-3 (Gal3), has been implicated in a variety of inflammatory and oncogenic processes, including tumor growth, invasion, and metastasis. The interactions of Gal3 and MUC16 represent a potential targetable pathway for the treatment of MUC16-expressing malignancies. We found that the silencing of Gal3 in MUC16-expressing breast and ovarian cancer cells in vitro inhibited tumor cell invasion and led to attenuated tumor growth in murine models. We therefore developed an inhibitory murine monoclonal anti–Gal3 carbohydrate-binding domain antibody, 14D11, which bound human and mouse Gal3 but did not bind human Galectins-1, -7, -8 or -9. Competition studies and a docking model suggest that the 14D11 antibody competes with lactose for the carbohydrate binding pocket of Gal3. In MUC16-expressing cancer cells, 14D11 treatment blocked AKT and ERK1/2 phosphorylation, and led to inhibition of cancer cell Matrigel invasion. Finally, in experimental animal tumor models, 14D11 treatment led to prolongation of overall survival in animals bearing flank tumors, and retarded lung specific metastatic growth by MUC16 expressing breast cancer cells. Our results provide evidence that antibody based Gal3 blockade may be a viable therapeutic strategy in patients with MUC16-expressing tumors, supporting further development of human blocking antibodies against Gal3 as potential cancer therapeutics.

## Introduction

Galectin-3 (Gal3) is a member of the family of small, highly conserved eukaryotic lectins that recognize specific complex sugars on glycosylated cell surface proteins. Galectin family members have complex biology, and family members can be found in the nucleus, cytoplasm, and pericellular space. Galectins have been implicated in the initiation of metastatic detachment from the primary tumor site, establishment of distant metastases through interactions with cell-surface glycoproteins, and promotion of metastatic tumor colonization through both their presence on metastasis-associated myeloid cells in the early metastatic niche and promotion of endothelial adhesion^1,2^. Extracellular galectins are primarily released via exosomes and do not appear to have the classic method of secretion from the endoplasmic reticulum or Golgi apparatus^3^.

Among galectins, Gal3 is unique in its structure, as it is the only chimeric galectin in humans. Gal3 inhibition has been proposed in a variety of inflammatory non-neoplastic diseases, including heart failure, pulmonary fibrosis, and steatohepatitis using small molecules, large glycosylated proteins and shRNA^4–6^. In the last decade it has also become apparent that Gal3 is important to link cancer cells with the stromal microenvironment. Gal3 modulates tumor cell development, adhesion, signaling, invasion, and immune system interaction^7–11^. Although Gal3 binds to other natural ligands, such as lactose, its highest affinity is for poly-*N*- acetyllactosamine chains, generated by the action of *N*-acetylglucosaminyltransferase V (Mgat5)^12^. Through binding and multimer formation through its amino terminal region, Gal3 forms linkages which regulate the position and surface residence time of growth factor receptors, including epidermal growth factor receptor (EGFR), platelet-derived growth factor receptor (PDGFR), integrins and cytotoxic T-lymphocyte membrane proteins^13,14^.

Mucin 16 (MUC16) is a complex, membrane-tethered mucin consisting of a small intracellular domain, hydrophobic transmembrane domain, 58 amino acid ectodomain proximal to the putative cleavage site, and a large, heavily glycosylated region of 156 amino acid tandem repeats that includes the CA125 epitope^15^. Given the oncogenic links of MUC16 and its tissue limited expression, several early-phase agents targeting MUC16 are being developed and tested^16–18^. In our previous work, we have focused on post-translational modification of the 58 amino acid MUC16 “ectodomain” at two proximal *N*-glycosylation sites (in classical Asn-X-Ser/Thr sequences), which act as potential drivers of oncogenic behavior^19,20^. Once expressed on the cell surface, the tetra-antennary N-glycan complexes (generated by the action of Mgat5 in the Golgi) serve as high-affinity binding partners for Gal3^21,22^. We have previously demonstrated co-immunoprecipitation of Gal3, MUC16, and Epidermal Growth Factor receptor (EGFR) protein and showed co-localization of the three proteins in ovarian cancer explants and human ovarian cancer sections^23^. Binding with Gal3 multimers, MUC16 can form stable signaling complexes with EGFR and Integrins, to enhance “outside in” signals. We have shown that oncogenic properties of ovarian cancer cell lines are increased by expression of N-glycosylated MUC16 on the cell surface^20^. Activation of stabilized receptor signaling leads to MUC16 dependent activation of SRC, ERK, and AKT, driving the production of key molecules involved in metastasis and invasion^24^. MUC16 expression depends on the Gal3 pathway to enhance tumor growth. This therefore represents a rational target for therapeutic applications in MUC16-expressing cancers.

Although several small molecule inhibitors of Gal3 are in development for treatment of diseases like melanoma and liver cirrhosis, their utility in malignancy is just beginning to be described^25,26^. To define the role of Gal3 in MUC16-dependent cellular activity, our lab previously created Gal3 knockdown cell lines from MUC16 overexpressing A2780 and SKOV3 cell lines (sh*LGALS3*-A2780 and sh*LGALS3*-SKOV3)^23^. These cell lines, with forced expression of the terminal amino acid sequence of the C-terminal MUC16 ectodomain, contain the *N*-glycosylation sites that drive tumor growth and invasion. In the previously published report, we used a blocking fusion protein engineered by fusing the sugar-binding domain of Gal3 with a truncated human IgG1 domain. This inhibits Gal3 pentamerization, which is necessary for its effects on oncogenic signaling. This dummy receptor, but not the engineered negative control, inhibited invasion in transfected SKOV3-MUC16 and A2780-MUC16, and also in OVCAR3, OVCA-432, OVCA-433, and CAOV3 cells. We further showed that a similarly constructed galectin-1 fusion protein did not demonstrate these inhibitory effects. In addition, we observed that loss of *LGALS3* expression inhibited MUC16-dependent phosphorylation of AKT, EGFR, and ERK1/2. Therefore, we hypothesized that therapeutic targeting of the Gal3 carbohydrate-binding domain would downregulate cancer cell invasion and growth.

In this report, we sought to develop high-affinity monoclonal anti–Gal3 antibodies directed at the Gal3 carbohydrate-binding domain and, using in vitro and in vivo models, to determine whether the interaction between MUC16 and Gal3 represents a potential targetable pathway for the treatment of MUC16-expressing malignancies.

## Results/discussion

### Gal3 expression in human high grade serous ovarian cancers

**Figure 1:**
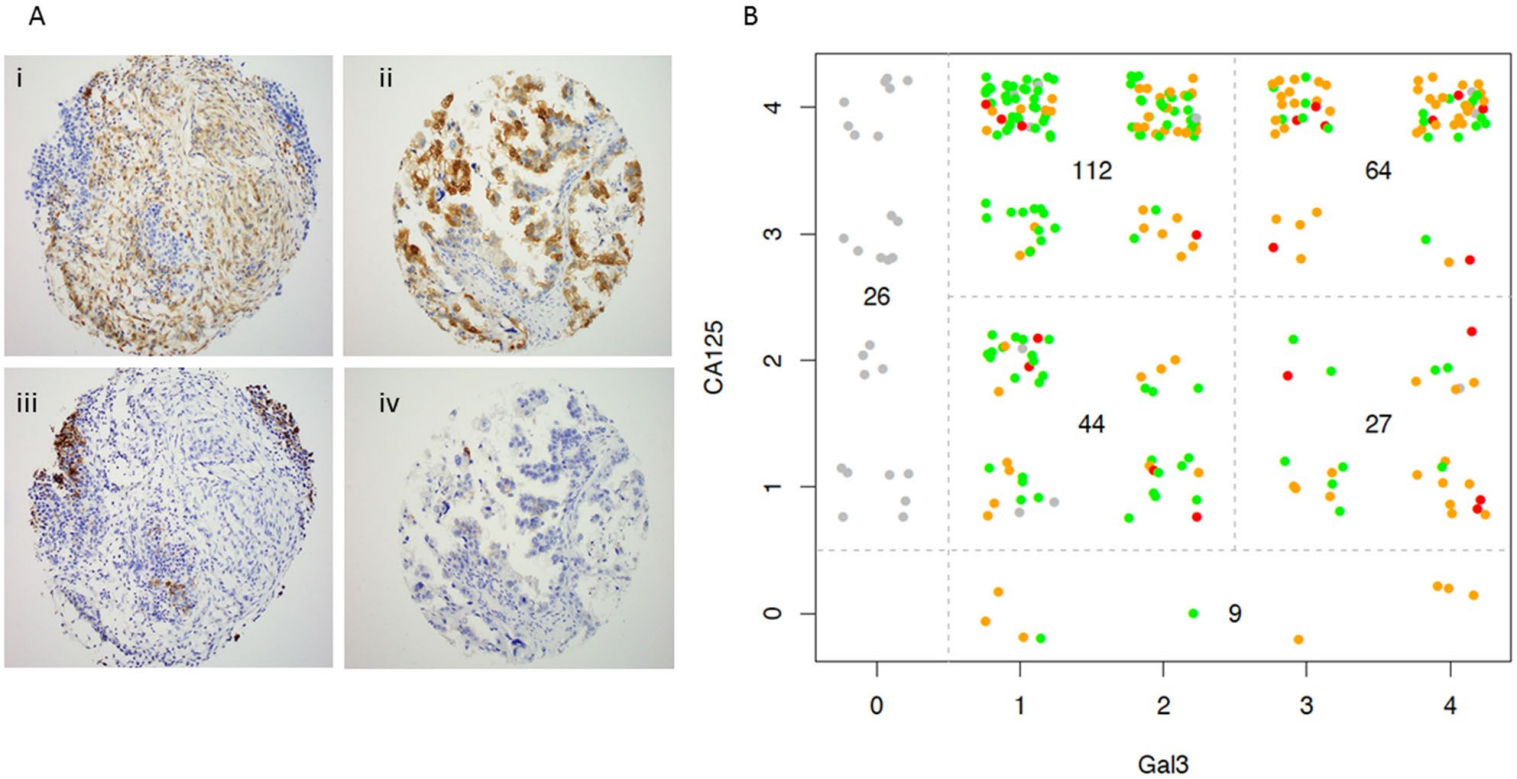
Expression of *LGALS3* and CA125 (MUC16) in human ovarian cancers. Three anonymized human tissue microarrays were constructed by collecting 1–3 cores from paraffin embedded, fixed blocks, as previously described^27^. (**A**) Examples of human tumor cores stained for CA125 (panel i) and Gal3 (panel ii). Examples of core that did not stain for CA125 (panel iv) and tumor that did not stain for Gal3 (panel iii). (**B**) Each core was qualitatively scored for prevalence of CA125 and Gal3 positive cells (0 =  < 5%, 1 +  = 5–25%; 2 +  = 25–50%; 3 +  = 50–75% and 4 +  =  > 75%. The intensity for Gal3 was separately scored as low, medium and high (represented by green, orange and red dots respectively). All cores were independently scored by a blinded reference pathologist (KP).

In our previous work we established the co-localization of MUC16 and Gal3 in cell lines, as well as in five ovarian cancers specimens^23^. In order to better establish the prevalence of co-expression of these proteins, we undertook a more robust survey by using previously developed immunohistochemistry techniques for Gal3 and CA125 to study 3 separate tissue microarrays from HGSOC tumor resections (examples in Fig. 1A)^27^. One to three cores (from different paraffin blocks) were included for each patient for a total of 282 cores with tumor tissue. Both the % of positive cells and the intensity of expression were scored by a blinded reviewer (Fig. 1B). Both CA125 and Gal3 were expressed in 247 of 282 sections (87% co-expression), while 26 cores had no Gal3 expression and 9 cores lacked CA125 expression but had some Gal3 protein present, making the Gal3 expression detectable in 90.7% of the human HGSOC cores examined. Both the % of positive cells and the intensity of expression varied across the tumor samples, but Gal3 expression significantly correlated with CA125 expression (Spearman's rank correlation rho = 0.168, *p* = 0.003), implying possible co-regulation of these proteins but without a strong correlation between intensity of expression for the two proteins.

### Gal3 knockdown model

**Figure 2:**
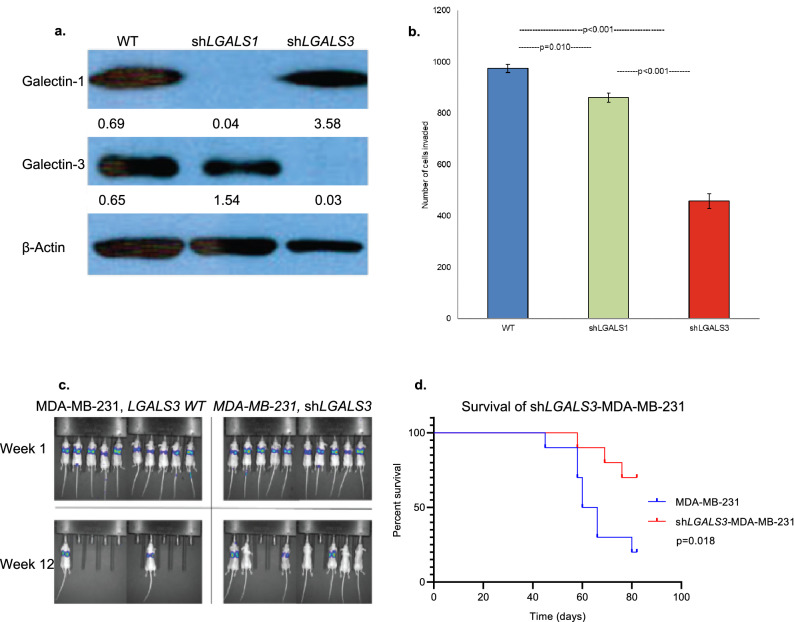
Knockdown sh*LGALS3-*MDA-MB-231 cells show decreased invasiveness both in vitro and in vivo*.* (**a**) *LGALS3* knockdown cell line, sh*LGALS3*-MDA-MB-231 and a similar *LGALS1* knockdown, sh*LGALS1*-MDA-MB-231 were generated. *LGALS3* and *LGALS1* silencing was confirmed by Western blot in a manner previously described^23^. β-Actin normalized densitometry quantification values are shown below each Western blot band. (**b**) In a Matrigel assay using triplicate chambers with 1 × 10^4^ cells/chamber, invasion of the sh*LGALS3*-MDA-MB-231 and sh*LGALS1*-MDA-MB-231 were compared to the parental MDA-MB-231 wild-type cell line after 48 h (*p* = 0.005, unpaired *t* test; error bars represent standard error). Three replicates were performed. (**c**) Representative luminescence images of all mice inoculated intravenously (by tail vein) with 5 × 10^6^ sh*LGALS3*-MDA-MB-231 cells or wild-type MDA-MB-231 control cells as previously described^31^. (**d**) Kaplan–Meier survival curves; median survival for mice (n = 10 female athymic nude mice) implanted with MDA-MB-231 wild-type cells was 60 days (95% CI, 53.8–66.2), while median survival for mice (n = 10 female athymic nude mice) implanted with shLGALS3-MDA-MB-231 cells was not reached (*p* = 0.02, log-rank test).

Having established high frequency for MUC16 and Gal3 co-expression, we explored the role of Gal3 in tumorigenicity and invasion. We have previously demonstrated that clinical biopsies of some breast cancers express high levels of MUC16^27^. The MDA-MB-231 cell is a breast cancer cell line derived from pleural effusion, which has been established by Massague et al*.*, as a model of organ specific tumor metastasis^28,29^. Given the high level of MUC16 expression, MDA-MB-231 cells were used as a model in the current study and an *LGALS3* knockdown cell line, sh*LGALS3*-MDA-MB-231, was generated^30^ (Fig. 2a). Similarly, an *LGALS1* knockdown cell line, sh*LGALS1*-MDA-MB-231, was created as a control to compare the different galectin targeting strategies (Fig. 2a). Using Matrigel invasion assays, we observed a significant decrease in the invasion of sh*LGALS3*-MDA-MB-231 cells compared to the parental wild-type cell line. Targeting of *LGALS1* in the MDA-MB-231 cells was inhibitory, but substantially inferior to targeting of *LGALS3* (*p* = 0.01, *p* < 0.001; Fig. 2b).

To examine the effects of *LGALS3* knockdown in vivo*,* athymic nude female mice were inoculated via tail vein injection with either MDA-MB-231 parental wild-type cells (n = 10) or sh*LGALS3*-MDA-MB-231 (n = 10)^29^. There was a significant delay in tumor growth in the animals inoculated with the knockdown cell line compared to controls over 12 weeks. At experiment conclusion, 2 of 10 mice in the wild-type cell line group were alive, both with disease, while 7 of 10 mice in the sh*LGALS3* group were alive, 4 with disease (Fig. 2c). Median survival for mice with MDA-MB-231 wild-type cells was 60 days (95% CI, 53.8–66.2), while median survival for mice implanted with sh*LGALS3*-MDA-MB-231 cells was not reached (*p* = 0.018) (Fig. 2d). Overall, these findings warranted additional studies targeting Gal3 as a therapeutic strategy, and we therefore set out to isolate Gal3 antibodies, particularly focused on binding to the carbohydrate binding domain.

### Generation of monoclonal anti-Gal3 antibodies

**Figure 3:**
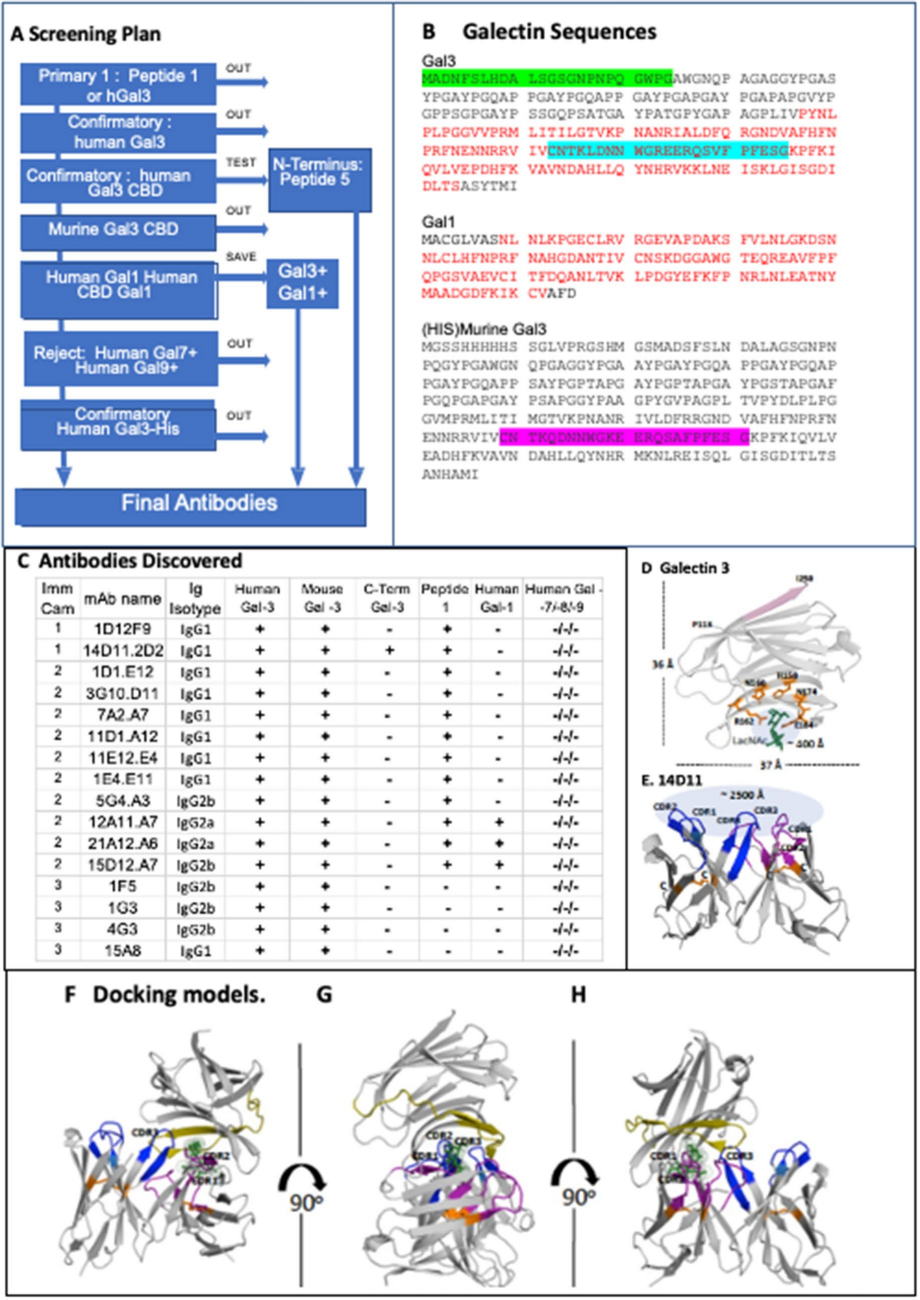
Generation of anti-Gal3 monoclonal antibody. (**A**) ELISA screening strategy for selecting candidate antibodies. The desired antibody screened positive for human Gal3, Peptide 1, his tagged Gal3, and mouse Gal3. It screened negative for human Gal 1, 7, 8, 9. A second antibody targeting N-terminus screened positive for human Gal3 and Peptide 5. (**B**) Galectin sequences. The carbohydrate binding domain is in red, the 23 amino acid sequence of the CBD that is conserved among species is highlighted in blue. Murine homolog is highlighted in magenta. Peptide 5, the N-terminus sequence, is highlighted in green. (**C**) Antibodies generated from campaigns 1, 2, and 3. (**D**) Gal3 crystal structure with N-acetyllactosamine (PDB: 4XBN), pink color: the last 6 amino acids (245-ASYTMI -250) which were omitted for 14D11 antibody screening, 5 amino acids (H158, N160, R162, N174, and E184) are participated in the LacNAc binding and green color is LacNAc. The solvent accessible area of sugar-binding pocket is approximately ~ 400 Å. (**E**) Computationally predicted V_H_ and V_L_ domain structure of 14D11, blue colors: 3 CDR regions of V_H_, purple colors: 3 CDR regions of V_L_, each of V_H_ or V_L_ has one disulfide bridge (orange color: Cys-Cys) and the solvent accessible area is approximately ~ 2500 Å. (**F**–**H**) The predicted docking model between Gal3 and 14D11 variable regions with 90 degree rotations, suggesting (1) the variable regions of 14D11 is positioned at a sugar binding pocket and (2) CDR3 of V_H_ and CDR1, CDR2 of V_L_ are occupying the carbohydrate-binding. pocket. Olive color of Gal3: peptide 1 regions (aa: 173-CNTKLDNNWGREERQSVFPFESG-195), which are conserved and a part of the sugar binding pocket.

The characteristics of desired monoclonal antibodies against the Gal3 carbohydrate binding domain (CBD) are illustrated in the flow chart in Fig. 3A. The optimal antibody would bind the human CBD (shown in red letters on Fig. 3B). Others have described the interactive sequence of the Gal3 CBD as a 23 amino acid sequence in the center of the CBD, highlighted in blue on Fig. 3B, which we designated as Peptide 1. The murine homolog is highlighted in magenta for comparison (Fig. 3B). Cross-reactivity with mouse Gal3 (mGal3) would be experimentally useful for validation of anti-tumor activity. The ideal antibody would not bind to other common galectins, and we used human Galectin-1, Galectin-7, Galectin-8, and Galectin-9 to identify and exclude cross-reactive antibody candidates. Finally, we were concerned about the complex chimeric binding properties of Galectin-3 and chose an NH2 His-Tag Gal3 protein as our final screen for CBD directed antibodies. In parallel, we also screened antibodies against the 23 amino acids at the NH2 terminus of Gal3. This sequence, highlighted in green, was designated as peptide 5.

In order to target the CBD, we began with the whole Gal3 protein immunization and gradually progressed to isolation of more specific antibodies according to the schedule in the supplement (Supplemental Table 1). Multiple clones secreting the desired antibodies were isolated from hybridomas, screened and sorted for reactivity to C-terminus or N-terminus of human Gal3 by immunosorbent assay (ELISA) leading to the generation of antibodies as described in Fig. 3C. Of note, because of the complexity of Gal3 binding behavior, we chose to use binding to a commercial NH2 His-Tag Gal3 protein as our final characterization step^32,33^. Monoclonal antibody (MAb) from only one clone met all of the selection criteria for further characterization: 14D11.2D2 (shortened, 14D11). The antibody isotype is an IgG_1_ with κ light chain. The 14D11 antibody bound to human and mouse Gal3, and Peptide 1, but not to human galectin-1, 7, 8, or 9 (Fig. 3C). The binding constant of 14D11 to Gal3 was evaluated by surface plasmon resonance (SPR)^34^. The 14D11 and 22417a.1D1.E12 (1D1) antibodies were bound to an anti-mouse Fc surface and recombinant human Gal3 protein delivered across a variety of concentrations (Fig. S2A). The 14D11 dissociation constant (K_D_), as measured by SPR, was 14.6 nM (Fig. S2B).

In order to better understand the 14D11-Gal3 interaction we also examined a docking model, following recent methods utilizing deep learning-based servers^35,36^. The variable domains of 14D11 showed a ~ 85% high sequence identities with template protein data blanks (PDBs), assumed to be folded into a very similar structure compared with a real structure. 14D11′s binding position as close to sugar-binding pocket area (Fig. 3F–H). The crystal structure of Gal3 CBD (PDB: 4XBN) showed that 5 amino acids within the peptide 1 sequence, including H158, N160, R162, N174, and E184 (which potentially form hydrogen bonds with N-acetyllactosamine (LacNAc)) which showed ~ 400 Å solvent-accessible area at the carbohydrate binding pocket (Fig. 3D). The area at the 6 complementarity determining region of 14D11 can cover up to ~ 2500 Å, resulting in 14D11 entirely occupying the CBD (Fig. 3E). Furthermore the K_D_ between Gal-3 CBD and 14D11 was ~ 14.6 nM, which represents ~ 13,000 times higher binding affinity compared with LacNAc’s K_D_ of ~ 0.2 mM. Lastly, the Peptide-1 designed for generating 14D11 antibody included two amino acids, N174 and E184, which form hydrogen bonds with LacNAc, suggesting that 14D11′s binding position could be close to the carbohydrate-binding pocket area (Fig. 3F–H). Taken together, our predictive docking models support the binding of 14D11 at the Gal3 CBD and provide a better understanding of the 14D11 mechanism, binding and inhibition of carbohydrate binding to Gal3.

### Functional 14D11 antibody interactions with Gal3 in vitro

**Figure 4:**
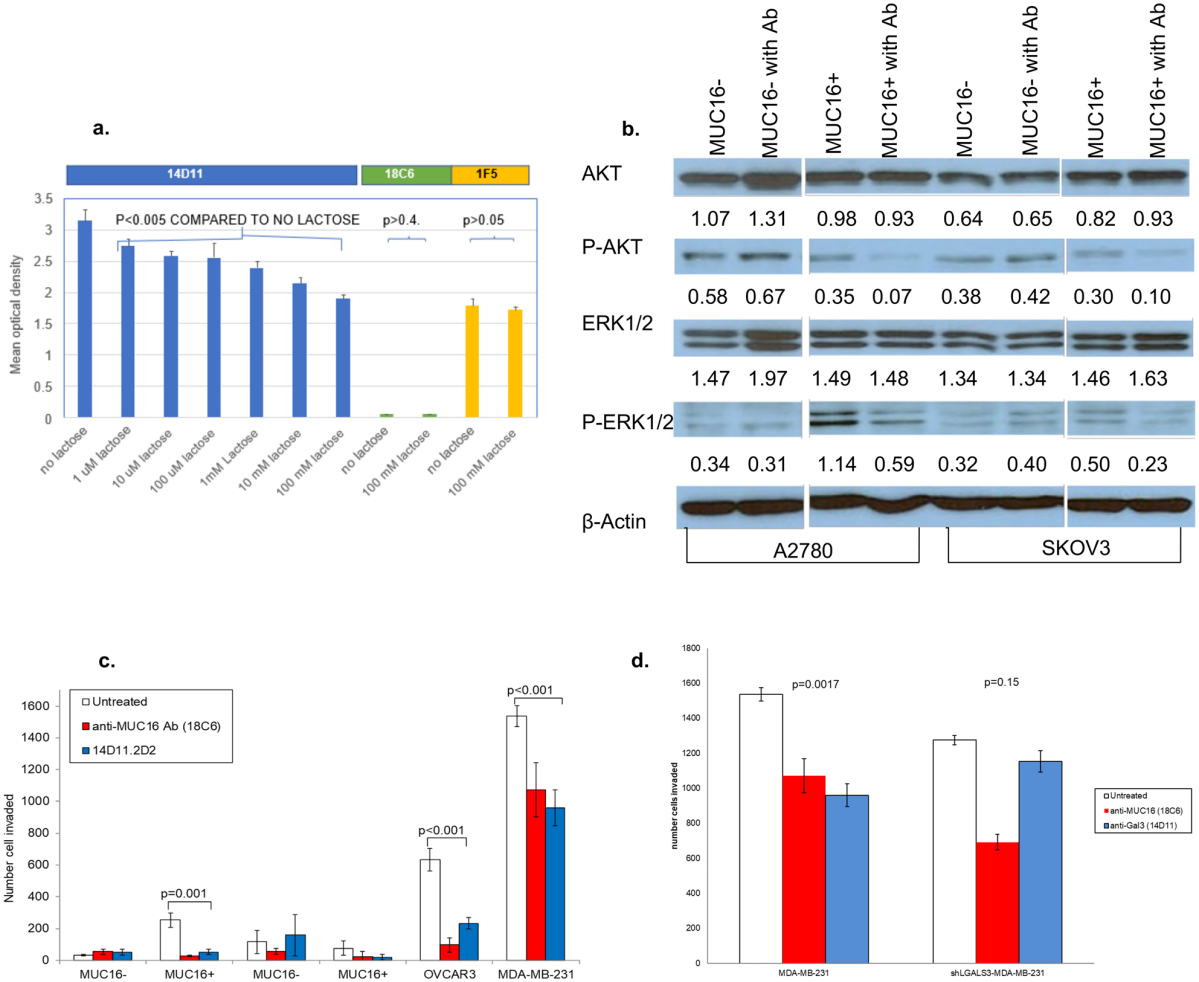
Anti-Gal3 monoclonal antibody, 14D11, inhibits MUC16-mediated tumorigenesis in vitro. (**a**) Direct ELISA for Gal3 binding to selected antibodies in the presence or absence of lactose. The presence of increasing lactose concentrations progressively inhibited the binding of Gal3 to 14D11, an isotype control antibody (18C6) and 1F5, an antibody reactive to the N terminal 15 amino acids of Gal3. Each lactose condition was compared to the zero lactose condition for the same antibody by paired t-test. (**b**) Effect of 14D11 on MUC16-mediated activation of ERK/AKT signaling in MUC16-expressing A2780^c344^ and SKOV3^c344^ cells after 48-h exposure to antibody. MUC16-negative A2780 and SKOV3 cells were used as controls. Densitometry was used to normalize the results against β-actin. (**c**) Effect of 14D11 on Matrigel invasion in MUC16-overexpressing SKOV3^c344^ and A2780^c344^ cells, and endogenous MUC16-expressing OVCAR3 cells. Anti-MUC16 N-glycosylation site antibody, 18C6, was used as a positive control. The values are the mean of triplicate well from a representative study and the invasion assays were repeated independently ≥ 3times. (**d**) Effect of 14D11 on Matrigel invasion in MDA-MB-231 and sh*LGALS3*-MDA-MB-231 cells, performed as in 3C.

We reasoned that a binding epitope involving portions of the carbohydrate binding domain implied that natural ligands, such as lactose, might compete with antibody binding. As shown in Fig. 4a, increasing concentrations of lactose induced a concentration dependent decrease in 14D11 binding to human Gal3. Lactose at 100 mM did not significantly alter binding between Gal3 and an isotype control antibody, 18C6, nor between Gal3 and antibody 1F5, which was selected for its binding in the final 23 amino acids of the Gal3 protein.

To examine the effect of the 14D11 antibody on cellular MUC16-mediated signaling, several strategies were used. As we have previously described, the interaction of MUC16 *N*-glycans with galectins on the cell surface membranes stabilizes cell surface receptors like EGFR, leading to activation of AKT and ERK^23^. To examine the effect of Gal3 blockade on MUC16-mediated ERK and AKT activation, MUC16-expressing A2780^c344^ and SKOV3^c344^ cell lines and the matched MUC16 negative controls were exposed to 14D11 for 24 h. These cell lines were examined for MUC16 and Gal3 expression and the results are shown in Fig. S1. The forced MUC16 expression increased phosphorylation of both AKT and ERK over MUC16 negative parental lines. The antibody 14D11-mediated Gal3 blockade resulted in decreased phosphorylation of ERK1/2 and AKT in MUC16-positive cell lines (Fig. 4b). Phosphorylation was not affected by the antibody in MUC16-negative cell lines.

To examine the biologic significance of Gal3 blockade, the effect of 14D11 was evaluated in Matrigel invasion assays (Fig. 4c) using a previously generated anti-MUC16 *N*-glycosylation site antibody (18C6) as a positive control^23^. Treatment of cells for 48 h with 14D11 decreased MUC16-dependent cellular invasion in SKOV3 and OVCAR3 cell lines (*p* = 0.001, *p* < 0.001, respectively) (Fig. 4b). Similarly, following 48 h exposure to 14D11, the MUC16 positive MDA-MB-231 metastatic breast cancer cell line Matrigel invasion was decreased (*p* = 0.002) but not in the related Gal3 knockdown cell line, sh*LGALS3*-MDA-MB-231 (Fig. 4c), confirming Gal3 as the primary target of 14D11.

### Therapeutic efficacy of 14D11 based Gal3 inhibition in vivo

**Figure 5:**
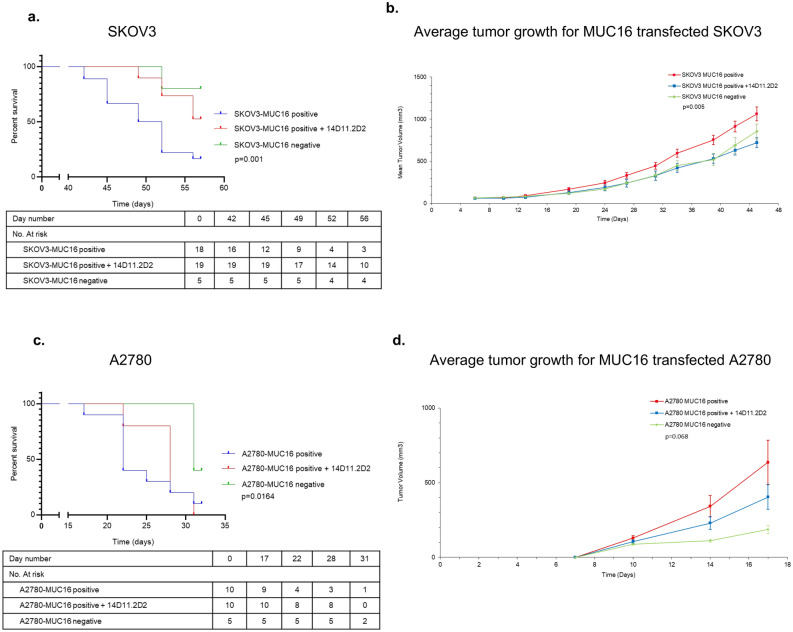
Therapeutic effects of Gal3 blockade in vivo. Kaplan–Meier survival curves for mice implanted with SKOV3^c344^, A2780^c344^, or MDA-MB-231 tumors are shown. (**a**) For mice implanted with SKOV3^c344^ tumors, the median survival time for the untreated group was 49 days (95% CI, 45.3–52.6), while the median survival for the group treated with 14D11 and for the group implanted with MUC16 negative SKOV3 cells was not reached after 56 days when the experiment was terminated (*p* = 0.001). (**b**) Average tumor growth for animals implanted with SKOV3 cells. (**c**) For the group implanted with A2780^c344^ cells, the median survival time for the untreated group was 22 days (95% CI, 19.6–24.4), while the median survival time for the group treated with 14D11 was 31 days (95% CI, not estimable), and median survival was not reached for animals implanted with MUC16 negative A2780 cells (*p* = 0.0164). (**d**) Average tumor growth for animals implanted with A2780 cells.

To examine the therapeutic effects of Gal3 blockade in vivo*,* the flanks of athymic nude mice each were implanted with 2 million cells subcutaneously using MUC16-expressing SKOV3^c344^ cells (SKOV3 MUC16 positive) and MUC16-expressing A2780^c344^ cells (A2780 MUC16 positive), as well as SKOV3 and A2780 wild type cells (SKOV3 MUC16 negative and A2780 MUC16 negative). In the SKOV3^c344^ experiment, of the 40 mice implanted with tumor cells, 3 showed no tumor growth after 7 days of implantation—1 in the treatment group and 2 in the control group. These mice were excluded from the subsequent analyses. Five mice were also implanted with SKOV3 MUC16 negative cells, as an untreated control. The median overall survival for the untreated group was 49 days (95% CI, 45.3–52.6), while the median survival for the group treated with 14D11 and for the group implanted with SKOV3 MUC16 negative cells was not reached after 56 days (*p* = 0.001) (Fig. 5a). Figure 5b depicts average tumor volume for the three groups. The average tumor volume was calculated at twice weekly intervals until the first mouse reached maximum IACUC permitted tumor volume or ulceration of the tumor requiring euthanasia (at day 45). Average tumor volume was significantly greater for mice implanted with SKOV3^c344^ that were not treated with antibody. These findings were further corroborated in the A2780^c344^ model: 20 mice were implanted with tumor cells, half of which were treated with 14D11. As a control, an additional 5 mice were implanted with A2780 MUC16 negative cells. Therapy with 14D11 decreased tumor growth when compared to the untreated MUC16 positive group, with a median survival of 22 days (95% CI, 19.6–24.4) in the untreated group and 31 days (95% CI, not estimable) for the 14D11 treatment group, while the A2780 MUC16 negative group did not reach median survival after 31 days (*p* = 0.0164) (Fig. 5c). Average tumor volume was also plotted for the three groups, with measurements taken at biweekly intervals until the first mouse reached maximum tumor volume or ulceration (17 days). The average tumor volume for mice untreated with antibody with A2780^c344^ cell approached statistical significance compared to mice treated with antibody and mice implanted with A2780 MUC16 negative cells (Fig. 5d).

**Figure 6:**
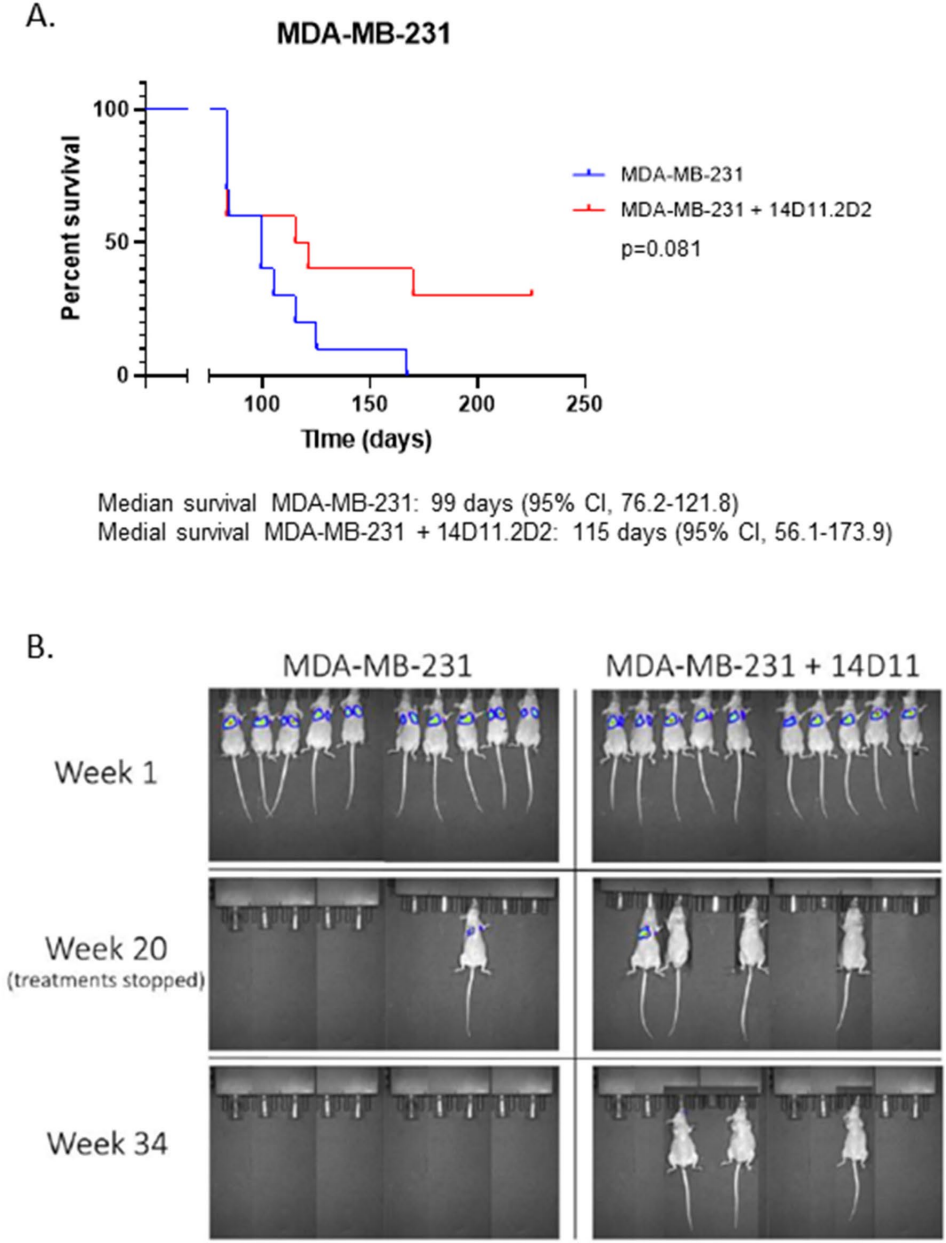
Therapeutic effects of Gal3 blockade on tumor metastases. (**A**) For the mice implanted with MDA-MB-231 cells, median survival for the untreated group was 99 days (95% CI, 76.2–121.8), while for group treated with 14D11, median survival was 115 days (95% CI, 56.1–173.9) (*p* = 0.081). (**B**) Representative luminescence images of mice at 1, 20, and 34 weeks.

Since sh*LGALS3* inhibited metastases as shown in Fig. 2, the effects of 14D11 on metastasis development was also explored. Twenty athymic nude mice were inoculated intravenously (IV) via tail vein injections with MDA-MB-231 cells. After 1 week, the presence of tumor cells in the lungs of all mice was evident on imaging. Half of the animals were then treated with 14D11 at a dose of 50 µg IV per mouse twice weekly, and the other half were treated with phosphate-buffered saline (PBS) as a control. Treatments were stopped at 20 weeks. Although there was prolongation in overall survival in the animals treated with 14D11, it did not reach statistical significance at the *p* < 0.05 level, likely due to the modest number of animals used and the high initial tumor load. (*p* = 0.08) (Fig. 6A,B).

## Conclusions

We hypothesized, based on our prior observations, that antibody directed at the Gal3 CBD would interfere with binding of Gal3 to complex *N*-glycoproteins like MUC16, resulting in inhibition of tumorgenicity. We were able to confirm that co-expression of MUC16 and Gal3 present in over 90% of the HGSOC core studied, and that Gal3 knockdown strategies had tumor inhibitory effects. Based on these observations we identified anti-Gal3 antibodies, including one antibody, 14D11, which competes for binding at the CBD site with lactose. A docking model confirms that the 14D11 interacts with Gal3 amino acids in the CBD. We demonstrated that antibody-mediated Gal3 blockade results in the predicted inhibition of tumor signaling pathways and tumor cell invasion, with evidence of anti-tumor activity observed in animal tumor models expressing MUC16 as a candidate glycoprotein partner. We have shown that murine monoclonal anti-Gal3 antibody, 14D11 disrupts MUC16-mediated growth invasion and oncogene activation.

Gal3 occupies a key position as a mediator of many inflammatory processes and is clearly linked to essential processes in cancer biology. Other common cancer-related mucins also have implicated Gal3 in their adverse effects^37^. Surprisingly, the Gal3 knockout mouse has a modest phenotype with impaired inflammatory responses and glucose metabolism^38,39^. Small molecules, plant saccharides, and shRNA interventions have all been studied preclinically as Gal3 directed therapeutics^4,8,9,25,40^. The antibody described here has several potential advantages over many of the other proposed therapeutics, as both a tool and therapy: 1) it maintains very high affinity, 2) it has the favorable kinetics of monoclonal antibodies, and 3) the lack of expected intracellular effects in a target that may be localized in the nucleus, the cytoplasm, the cell surface, and the microenvironment. Taken together, the results of this study provide a rationale for the development of human anti–Gal3 antibodies for use in the treatment of ovarian cancer and other mucin-expressing tumors.

## Methods

### Immunohistochemistry

Immunohistochemistry was done on the tissue microarrays with both standard CA125 (Ventana, Tuscon,AZ) and mAb 9C4 (Cell Marque, Sigma-Millipore) according to our prior methods^27^. The CA125 staining was done precisely as our prior work. The Gal3 staining was performed at a dilution of 1:50 with a 30-min incubation time. All of the stained sections were reviewed by a reference pathologist (KJP). A subset of cores for which there was equivocal staining was also independently scored by a second pathologist to ensure consistency in scoring methods. Only cytoplasmic and/or membranous staining was considered positive. If a portion of the cell showed membranous staining, that was considered partial staining. A scoring system was devised to provide a semi-quantitative assessment of staining distribution and intensity in individual cores. At the same time, it was designed to be useful for comparing the staining distribution and intensity between CA125 and the anti-Gal3 antibody. The score incorporated the percentage of positive staining cells according to the following standards: score 0: no staining; score 1: 1–25% stained; score 2: 25% to 50% stained; score 3: 51% to 75% stained: score 4: 76% to 100% stained. The Gal3 staining was also scored by intensity 1 = weak; 2 = moderate, and 3 = strong. The pathologist first reviewed all tissue microarrays stained with CA125 and scored each core. Then, the same cores stained with the novel antibodies were scored 1 to several days after CA125 without reference to the earlier results. Direct comparison of the scoring between the stains for each core was made only after all of the scoring was completed. The results were placed in the figure based on pattern of staining and the intensity of Gal3 staining recorded from 1–3 with green, orange and red colored dot marker, respectively.

#### Fluorescence activated cell sorting (FACS)

Flow cytometric analyses were performed using Gallios Flow Cytometer with Kaluza soft- ware (Beckman Coulter, Brea, CA, USA). Cells were resuspended with the appropriate antibody, diluted in FACS buffer and incubated at 4 °C for 30 min in the dark. The cells were subsequently washed 3 times with cold FACS buffer and resuspended in 1X DAPI prior to FACS analysis.

### Antibody generation

Gal3 fusion proteins were generated using pFUSE-hIgG1-Fc2 vector (InvivoGen; San Diego, CA), which serves as a ‘dummy’ antibody with a human IgG1-Fc backbone^20,23^. PCR primers were designed with the restriction enzyme site EcoRV as the forward primer and NcoI as the reverse primer to amplify the DNA encoding a fragment of Gal3 sequence (*LGALS3*) (amino acids 117-244), which includes the *LGALS3* sugar-binding domain. A *LGALS3* cDNA clone (MGC:2058 IMAGE:3050135 GenBank: AAH01120.1; DBSource accession BC001120.2, obtained from ATCC [Manassas, VA]) was used as a template DNA to synthesize the sugar-binding domain of the *LGALS3* as a PCR product. The PCR product encoding ^117-*244*^*LGALS3* was then purified on a 1% agarose gel, sequenced, and inserted into the pFUSE-IgG1-Fc2 vector to generate ^117-*244*^*LGALS3*-pFUSE-hIgG1-Fc2. ^117-*244*^*LGALS3*-pFUSE-hIgG1-Fc2 fusion protein was expressed using the FreeStyle 293 Expression System, and the supernatant was purified using Protein-A column^20,23^.

To generate antibodies, 5 female BALB/c mice were immunized with a series of immunogens at a dose of 50 µg immunogen/mouse intraperitoneally (IP) every 3 weeks as detailed in the supplemental material (Supplemental Table 1). In Campaign 1, the first 5 immunizations were administered with the ^117-*244*^*LGALS3*-pFUSE-hIgG1-Fc2 fusion protein. The last 2 immunizations used human LGALS3 protein (OriGene; Rockville, MD). The immune sera from the mice were collected at the time of every immunization and screened for reactivity against LGALS3 by ELISA. Following the last immunization, 2 of the 5 immunized mice that showed high ELISA titers to the antigen were selected and IV boosted with the human LGALS3 protein at a dose of 10 µg immunogen/mouse (one mouse was planned to be sacrificed while the other was selected as a backup). The fusion and screening was performed according the protocols of the MSKCC monoclonal antibody facility and our prior reports^23,27^. To increase number of CBD directed antibodies, a second campaign was undertaken. A 23 amino acid custom peptide was synthesized from the sugar-binding domain of LGALS3, CNTKLDNNWGREERQSVFPFESG (called Peptide 1) (All peptides commercially supplied by Atlantic Peptides; Lewisburg, PA). The peptide sequence was chosen as it was noted to be conserved among various species and therefore likely represented a critical region of the protein. The peptide was then conjugated to keyhole limpet hemocyanin (KLH) using Imject Maleimide-Activated mcKLH Kit (Pierce; Rockford, IL), creating the KLH-Peptide 1. Four mice were immunized with KLH-Peptide 1 at 50 µg immunogen/mouse IP every 3 weeks for a total of 3 injections. Immune sera were collected and screened for reactivity against LGALS3 and Peptide 1 by ELISA. Following the last immunization, 2 mice were chosen to receive an IV boost of KLH-Peptide 1 at 10 µg immunogen/mouse, and 24 h later, one mouse was sacrificed. The splenocytes of this mouse were fused with a hybridoma fusion partner, according the protocols of the MSKCC monoclonal antibody facility. In a third campaign, the antibodies were produced by immunizing the mice with Peptide 1 and Peptide 5. Peptide 5 was a custom peptide similarly synthesized from the N-terminus of Gal3 (MADNFSLHDALSGSGNPNQGWPG; Atlantic Peptides; Lewisburg, PA). Peptide 9 was a custom peptide that was synthesized from the N-terminus of Gal3 (CMADNFSLHDALSGSGNPNQGWPG; Atlantic Peptides; Lewisburg, PA) which has a Cysteine at the N-terminus to conjugate with KLH as described above, creating the KLH-Peptide 9 used for immunization. The mice were screened similar to campaign 2 with the addition of being screened for reactivity against Peptide 5.

### Functional assays

Once the purified anti-Gal3 supernatants were obtained, they were screened for their activity using binding, invasion, and anti-tumor assays, as described in Fig. 3A. Antibodies deemed to be potentially useful were subcloned, retested and stored for future use.

#### ELISA

Purified antibodies that bound to Gal3 were confirmed by ELISA. For this, 2 strategies were used. The first utilized a plastic 96-well microplate coated with recombinant human Gal3 (rGal3) protein (Abcam; Cambridge, MA), synthetic peptide (Gal3 CBD—Peptide 1; Gal3 NH2 terminus—Peptide 5 ), Mouse Gal3, human Galectin-1 (Gal1), Galectin-7(Gal7), Galectin-8 (Gal8), or Galectin-9 (Gal9) (Sigma) and an isotype-specific secondary antibody conjugated Horseradish-Peroxidase (HRP) for detection. As there was concern that Gal3 would not retain its native confirmation on a plastic plate, for the second strategy, a Gal3 construct was created with polyhistidine-tag at the N-terminus (Abcam, ab89487). A nickel-coated 96-well microplate was used with polyhistidine-tagged Gal3 (his-Gal3) as the primary protein. Similarly, an isotype-specific secondary antibody was used for detection. Preferred antibody screened positive for rGal-3, his-Gal3, mouse Gal3, and Peptide 1. The antibody screened negative for human Gal1, Gal7, Gal8, and Gal9. Similarly, an antibody targeting N-terminus of Gal3 was generated. It screened positive for rGal-3 and Peptide 5.

#### Surface plasmon resonance (SPR)

Antibody binding to Gal3 was confirmed by SPR assay. The 14D11 antibody was captured on an anti-mouse Fc surface and an 8-pt, twofold dilution series of Gal3 was flown over the antibody starting at 500 nM. All values adjusted by baseline standardization and subtraction of a no ligand channel for all analyses. The output for 14D11 is included in Supplemental Fig. S2.

#### Western blot

Detailed methods were described in previous work^20,27^. The intact Western blot, before placement in Figs. 2 and 4 is shown as Supplemental Figs. S3, S4, respectively.

#### The predicted structure of 14D11 variable regions and a docking model

The variable regions of V_H_ (1Q-117S) and V_L_ (1D-107K) were predicted through a deep learning-based prediction server (https://github.com/gjoni/trRosetta) (Fig. 3E)^35,36^. The PDBs, 5KTE for V_H_ and 5KOV for V_L_, were used as model-templates with high sequence identities. The docking model was initiated using the Rosetta FlexPepDock (http://www.rosettacommons.org) which can predict their interaction through a hybrid docking algorithm. The top 3 models were analyzed and filtered according to the high scores. A final model for further analysis was carefully examined and the position was aligned with different views (Fig. 3F–H).

#### Cell lines

Several cell lines were used for the in vitro and in vivo experiments. SKOV3^c344^ and A2780^c344^: MUC16 ectodomain-expressing cell line. The original cell lines were secured from the the ATCC (ATCC.org). Briefly, the cells were created by transfecting MUC16-negative human ovarian cancer cell lines (SKOV3 and A2780) with the sequence elements of the C-terminal MUC16, using the phrGFP vector (A2780-phrGFP-MUC16^c344^, or A2780^c344^; SKOV3-phrGFP-MUC16^c344^, or SKOV3^c344^). OVCAR3 cells are a wild-type MUC16-expressing cell line initially derived from ascitic fluid^27,41^, obtained from the ATCC. A2780 and OVCAR3 cells were cultured in RPMI with 10 mM HEPES, 10% fetal calf serum (FCS), 2 mM L-Glutamine and 0.2 U/mL insulin, 100 U/mL penicillin, and 100 ug/mL streptomycin. SKOV3 cells were cultured in DMEM low glucose with 10% FCS, 4 mM L-Glutamine, 100 U/mL penicillin, and 100 ug/mL streptomycin^20,23,27^. The MUC16 ectodomain sequence on OVCAR3 as well as on transfected cell lines (SKOV3^c344^ and A2780^c344^) was recognized by the MUC16 ectodomain-specific antibody, 4H11, by FACS analysis (Supplemental Fig. S1). Similarly FACS analysis confirmed the presence of Gal3 on the three cell lines using the 14D11 anti-Gal3 antibody, as well as a commercial Gal3-APC antibody (R&D Biosystems, IC1154A) (Supplemental Fig. S1).

The MDA-MB-231 cell line is a wild-type breast cancer cell line that has been noted to express MUC16 and lead to lung metastases in mouse models, provided by Dr. Joan Massague from Sloan Kettering Institute^29,30^. Short-hairpin RNA *LGALS3* and *LGALS1* knockdown models of the MDA-MB-231 cell line were generated using a previously described protocol (sh*LGALS3*-MDA-MB-231 and sh*LGALS1*-MDA-MB-231)^27^. The knockdown was confirmed by Western blot of cell line lysates (Fig. 2a). MDA-MB-231 cells were cultured in DMEM high glucose with 10% FCS, 4 mM L-Glutamine, 2 mM sodium pyruvate, 100 U/mL penicillin, and 100 ug/mL streptomycin.

#### Matrigel invasion assay

Antibody inhibition of basement membrane invasion was determined in Matrigel invasion chambers, as previously described^20,23^. Briefly, the MUC16-transfected cells as well as wild-type MUC16-expressing cells were pretreated with a 50 µg of antibody prior to exposure to the Matrigel invasion chambers. Control chambers were set up with cells that had not been exposed to antibody. Each insert contained 10,000 cells per chamber. Both experimental and control chambers were set up in three biologic replicates. The number of invading cells was counted after a 48-h incubation period. Each study was replicated ≥ 3 times.

#### Mouse models

Mouse experiments were approved by the Memorial Sloan Kettering Cancer Center (MSK) Institutional Animal Care and Use Committee and all experiments were performed in accordance with relevant guidelines and regulations. The study was carried out in compliance with the ARRIVE guidelines. Lead antibody from the in vitro experiments was used to evaluate antibody efficacy in a transplanted ovarian tumor growth xenograft model. Initially, SKOV3-MUC16^c344^ cells were introduced into the flank area of female, athymic nude mice at 2 million cells per mouse. Twenty mice in the experiment group received twice weekly antibody injections at 50 µg/mouse IV, while an additional 20 in the control group were treated with PBS. All treatments were started on the same day as cell implantation. Five additional mice were implanted with wild type SKOV3 and kept as control. The experiment was repeated using A2780-MUC16^c344^ cell lines. Similarly, 2 million cells per mouse were introduced into the flank area of female, athymic nude mice (n = 20). Ten mice received twice weekly antibody injections, while 10 control mice received twice weekly PBS injections. An additional 5 mice were implanted with wild type A2780 and kept as control. Routine animal care was provided by the MSK Antitumor Assessment Core Facility. Tumor measurements were taken twice a week, and tumor growth was recorded per MSK Research Animal Resource Center guidelines. Animals were euthanized after reaching a maximum tumor volume of 1500 mm^3^ or if the tumor became ulcerated. As such, the SKOV3-MUC16^c344^ experiment was terminated after 56 days and the A2780-MUC16^c344^ experiment was terminated after 31 days, when the majority of mice had reached maximum tumor volume/met criteria for euthanasia. All mice, regardless of tumor size, were euthanized at this time.

A separate metastatic mouse model was conducted with tail vein inoculations of either 1.5 million MDA-MB-231 *LGALS3* wild-type or sh*LGALS3*-MDA-MB-231 cells in 200 µL of PBS/mouse into 10 and 10 (total 20) female, athymic nude mice, respectively. No further treatments were administered. Routine animal care, as outlined above, was conducted, and the mice were imaged once every 2 weeks with tumor luminescence using Living Image software (Living image Software version number : 4.3.1 URL: https://ctac.mbi.ufl.edu/files/2017/02/@-IVIS-Spectrum-User-Manual-4.3.1.pdf; PerkinElmer; Waltham, MA). The animals were euthanized for signs of illness, including poor mobility, decreased eating, and hunching, as defined by the MSK Research Animal Resource Center, with the dates of death recorded.

A second, antibody treatment based metastatic mouse model was conducted with tail vein inoculations of 1.5 million MDA-MB-231 cells in 200 µL of PBS/mouse into 20 female, athymic nude mice. A treatment group of 10 mice was administered twice weekly with antibody at a dose of 50 µg antibody/mouse in 200 µL of PBS by tail vein injection, and the control group received treatment with 200 µL of PBS. All treatments were started on the same day as cell implantation and stopped after 20 weeks of treatment. Routine animal care was conducted, and the mice were imaged once every 2 weeks with tumor luminescence, as outlined above. The animals were euthanized using health parameters, as outlined above.

### Statistical analyses

To compare studies of growth and invasion, data were expressed as mean ± standard error (SE) and analyzed for statistical significance using unpaired student’s *t* test. Kaplan–Meier curves were constructed for survival and log-rank test used to determine significance, with significance defined as *p* < 0.05 and confidence interval not intersecting 1. The relationship between CA125 and Gal3 staining was performed using a Spearman Correlation test. (GraphPad Prism version 8.00 for Windows, GraphPad Software, La Jolla CA USA, www.graphpad.com).

## Supplementary Information

Supplementary Information
