# Supplementary material for: Targeting galectin-3 with a high-affinity antibody for inhibition of high-grade serous ovarian cancer and other MUC16/CA-125-expressing malignancies
Source: Sci Rep. 2021 Feb 12;11:3718. doi: 10.1038/s41598-021-82686-3 (PMC7881041; doi:10.1038/s41598-021-82686-3)
Supplement: Supplementary file 1 — Supplementary Information [file 41598_2021_82686_MOESM1_ESM.pdf]

TARGETING GALECTIN-3 WITH A HIGH-AFFINITY ANTIBODY FOR INHIBITION OF HIGH GRADE SEROUS OVARIAN CANCER AND OTHER MUC16/CA-125–EXPRESSING MALIGNANCIES

Marina Stasenko,[1,2§] Evan Smith,[1,§] Oladapo Yeku,[4,5] Kay J Park,[1,6] Ian Laster[4], Kwangkook Lee[4,5,] Sven Walderich,[7] Elizabeth Spriggs,[8] Bo Rueda[9,10], Britta Weigelt,[1] Dmitriy Zamarin, [11,12] Thapi Dharma Rao,[3τ] David R Spriggs[4,5τ*]

[1]Gynecology Service, Department of Surgery, Memorial Sloan Kettering Cancer Center, New York, NY, 10065

[2]Division of Gynecologic Oncology, Department of Obstetrics and Gynecology, NYU Langone Health, New York, NY, 10016

[3]Department of Medical Oncology, Monoclonal Antibody Core, Dana-Farber Cancer Institute, Boston, MA, 02215

[4]Division of Hematology-Oncology, Massachusetts General Hospital, Boston, MA, 02114

[5]Department of Medicine, Harvard Medical School, Boston, MA, 02114

[6]Department of Pathology, Memorial Sloan Kettering Cancer Center, New York, NY 10065

[7]Department of Medicine, University of California San Francisco Medical Center, San Francisco, CA, 94143

[8]Elizabeth Spriggs, Arnold Arboretum, Boston MA, 02131

[9]Vincent Center for Reproductive Biology, Department of Obstetrics and Gynecology, Massachusetts General Hospital, Boston, MA, 02114

[10]Department of Obstetrics and Gynecology and Reproductive Biology, Harvard Medical School, Boston, MA, 02114

[11]Gynecologic Medical Oncology Service, Department of Medicine, Memorial Sloan Kettering Cancer Center, New York, NY, 10065

[12]Department of Medicine, Weill Cornell Medical College, New York, NY, 10065

[§]These two authors contributed equally to this work

τThese two authors contributed equally to this work

***Corresponding author:**
David R. Spriggs, MD
Director of Gynecologic Cancer Program
Massachusetts General Hospital
Harvard Medical School
55 Fruit St
Boston, MA 02114
646-787-5920
dspriggs@mgh.harvard.edu

TABLE OF CONTENTS

Figure S1 Expression of LGALS3 and CA125 (MUC16) in human ovarian cancer cell lines

Figure S2 Surface Plasmon Resonance study of Anti–Gal3 monoclonal antibody, 14D11, binding to Gal3

Figure S3 Validation of siRNA knockdowns for Galectin 1 and Galectin 3, including cropped (A) and original (Figure 2A) (B) Uncropped Western Blots.

Figure S4 Western Blots (FIGURE 4B) with (B) uncropped originals

Table S1. Immunization Schedules for each antibody campaign

SUPPLEMENTAL FIGURE LEGENDS

Supplemental Figure 1S. Expression of LGALS3 and CA125 (MUC16) in human ovarian cancer cell lines. OVCAR3 and SKOV3^{c344} cell lines were stained for extracellular MUC16 and intracellular LGAL3 and evaluated via flow cytometry.

Supplemental Figure S2. Anti–Gal3 monoclonal antibody, 14D11, demonstrates binding to Gal3. **A.** Binding of antibody to human galectin-3 (R&D Systems; Minneapolis, MN) by surface plasmon resonance (SPR; GE Biacore; Pittsburgh, PA) according methods described by Mehta-D'souza et al (33). The antibodies to be tested were bound to a gold chip activated with mouse Fc receptor. Galectin-3 (R and D Systems) was used as ligand and delivered at a constant flow rate. 1D1 was used as a isotype control antibody without desired characteristics. **B.** A series of increasing concentrations Gal3 and analysis to fit the binding curves. The dissociation constant for the 14D11 antibody was estimated as 14.6 nM.

Supplemental Figure S3: Uncropped Western Blots. A. Cropped Western Blot (from Figure 2A) confirming silencing of *LGALS3* in the *LGALS3* knockdown cell line, sh*LGALS3*-MDA-MB-231 and confirming silencing of *LGALS1* in the *LGALS1* knockdown cell line, sh*LGALS1-MDA-MB-231*. β-Actin normalized densitometry quantification values are shown below each Western blot band. **B.** Uncropped Western Blots

Supplemental Figure 4S: Cropped Western Blot (Figure 4B) showing the effect of 14D11 on MUC16-mediated activation of ERK/AKT signaling in MUC16-expressing A2780^{c114}, A2780^{c344}, SKOV3^{c114} and SKOV3^{c344} cells after 48-h exposure to antibody. MUC16-negative A2780 and SKOV3 cells were used as controls. β-Actin normalized densitometry quantification values are shown below each western blot band. **B.** Uncropped Western Blots

Supplemental Table 1: Immunization schedules used for the three separate immunization campaigns to generate anti-galectin 3 monoclonal antibodies.

Supplemental Figure 1:   Expression of MUC16 and Galectin-3

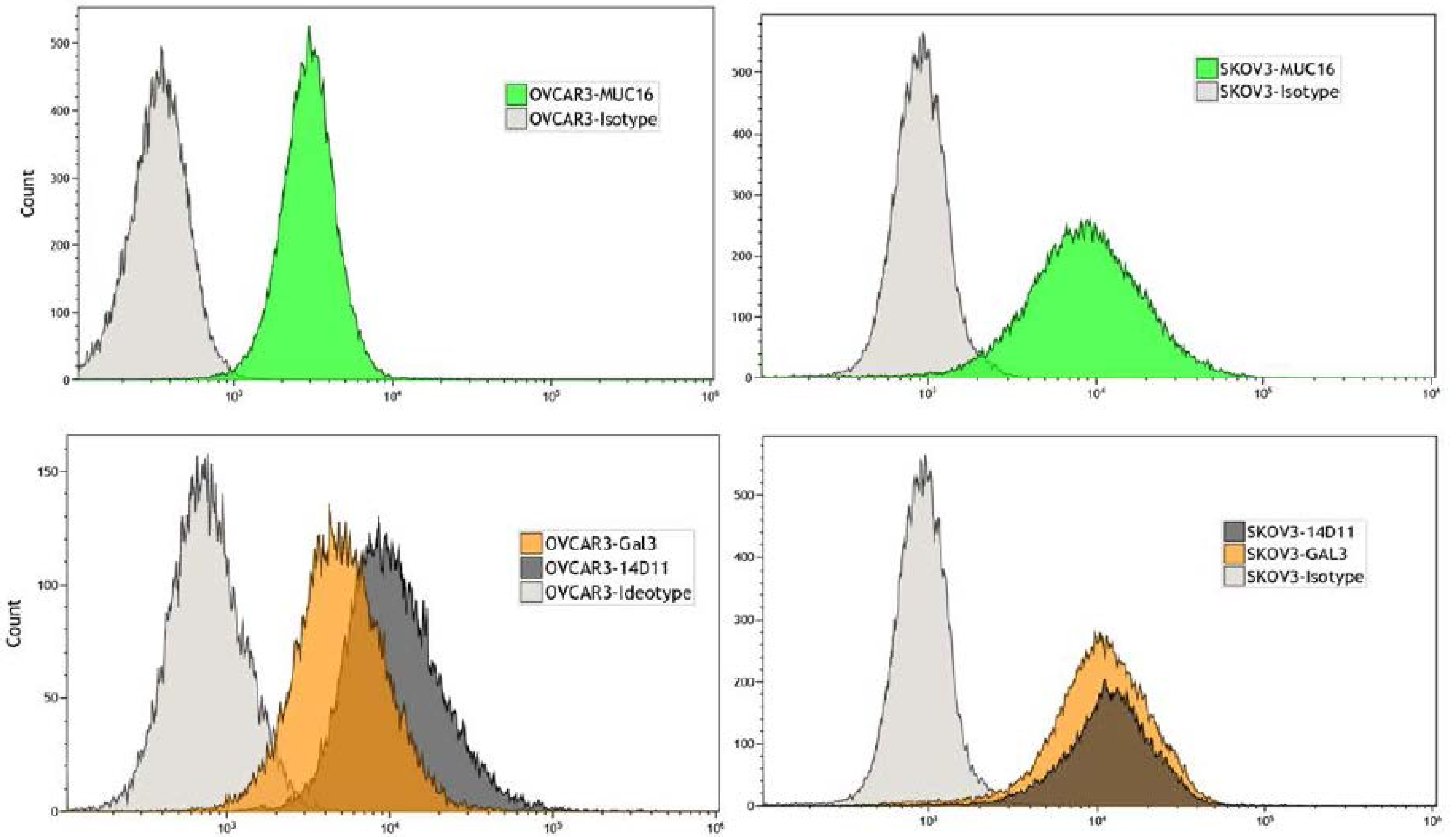

OVCAR3-MUC16
OVCAR3-Isotype
SKOV3-MUC16
SKOV3-Isotype
OVCAR3-Gal3
OVCAR3-14D11
OVCAR3-Ideotype
SKOV3-14D11
SKOV3-GAL3
SKOV3-Isotype
Count
Count

SUPPLEMENTAL FIGURE S2

A.

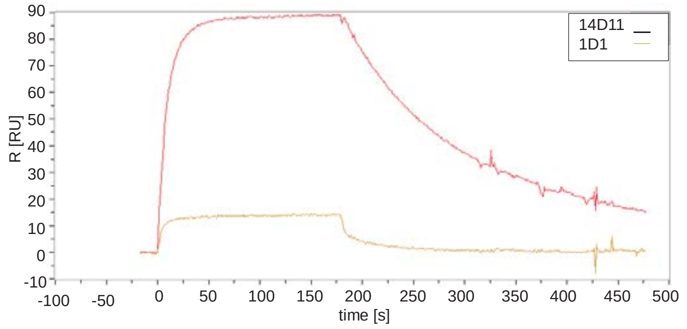

14D11
1D1
R [RU]
time [s]
90
80
70
60
50
40
30
20
10
0
-10
-100
-50
0
50
100
150
200
250
300
350
400
450
500

B.

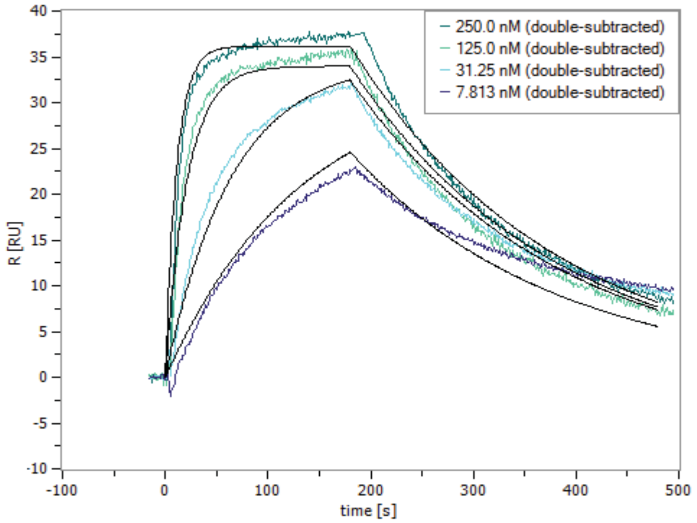

250.0 nM (double-subtracted)
125.0 nM (double-subtracted)
31.25 nM (double-subtracted)
7.813 nM (double-subtracted)
R [RU]
time [s]
40
35
30
25
20
15
10
5
0
-5
-10
-100
0
100
200
300
400
500

SUPPLEMENTAL FIGURE S3

A

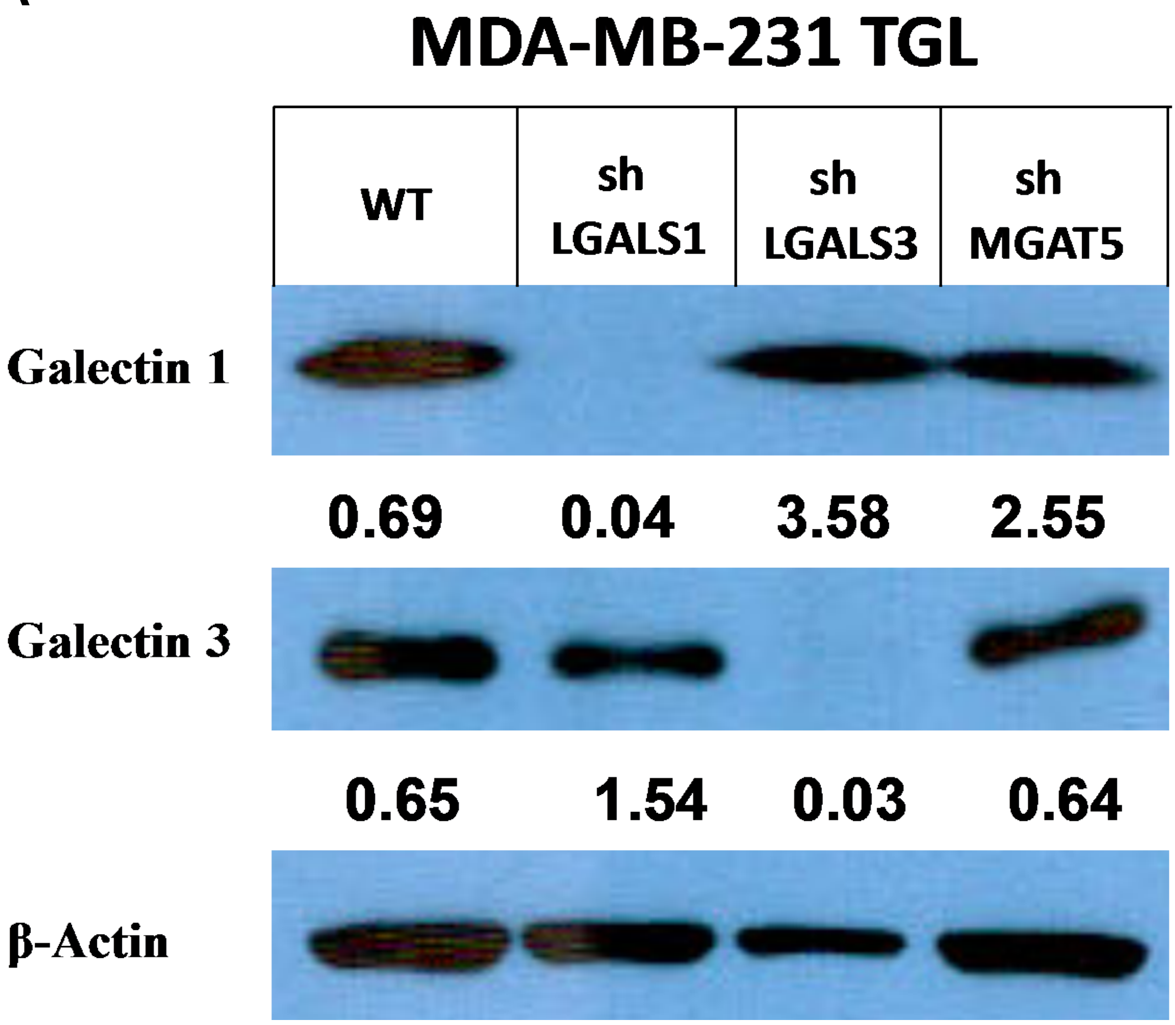
MDA-MB-231 TGL
WT
sh LGALS1
sh LGALS3
sh MGAT5
Galectin 1
0.69
0.04
3.58
2.55
Galectin 3
0.65
1.54
0.03
0.64
β-Actin

B

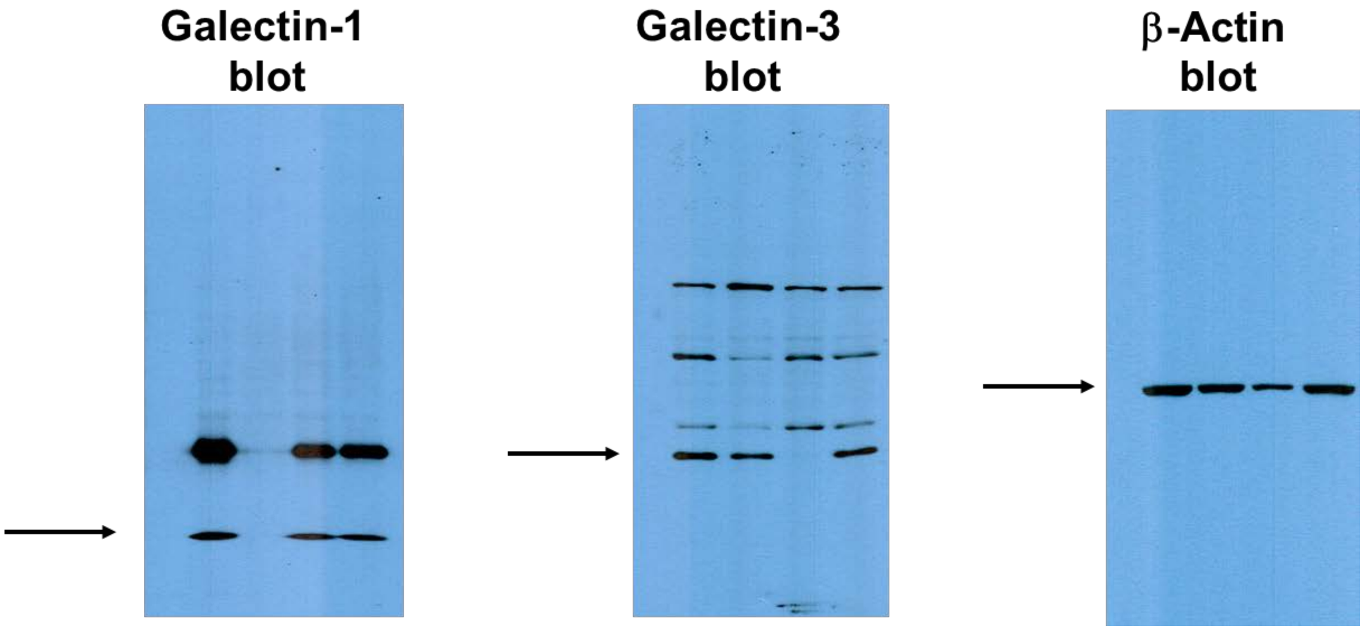
Galectin-1 blot
Galectin-3 blot
β-Actin blot

SUPPLEMENTAL FIGURE S4

A

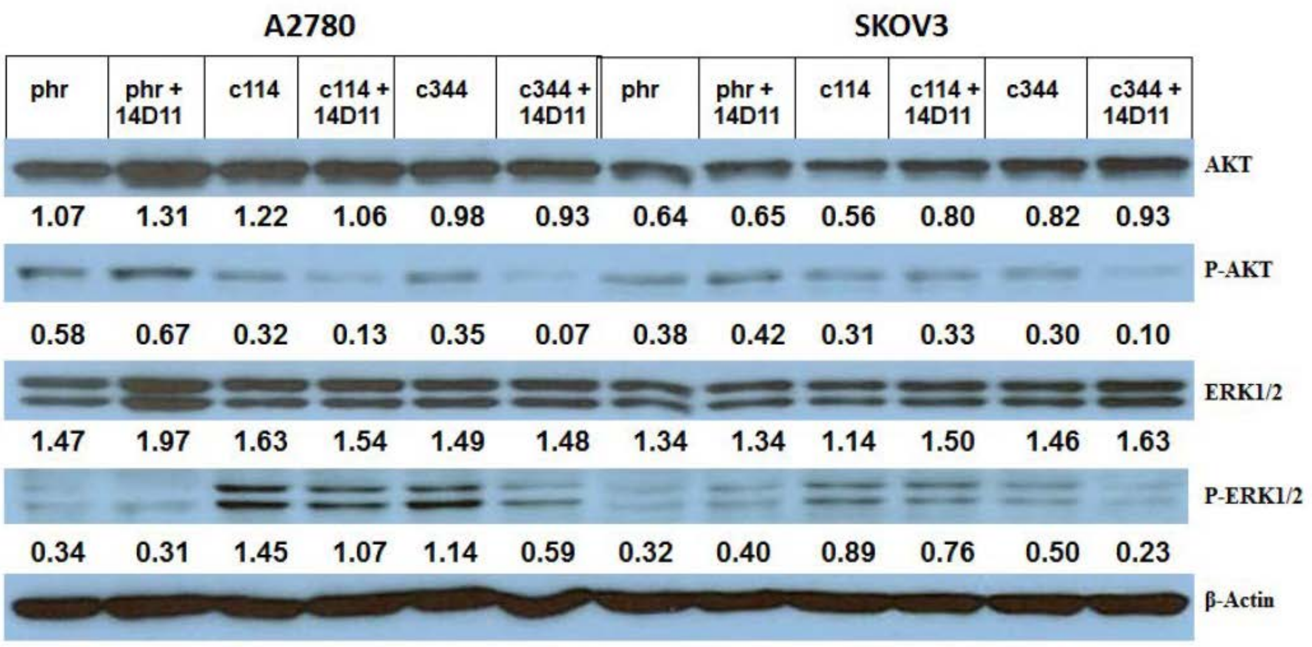
A2780
SKOV3
phr
phr + 14D11
c114
c114 + 14D11
c344
c344 + 14D11
phr
phr + 14D11
c114
c114 + 14D11
c344
c344 + 14D11
AKT
1.07 1.31 1.22 1.06 0.98 0.93 0.64 0.65 0.56 0.80 0.82 0.93
P-AKT
0.58 0.67 0.32 0.13 0.35 0.07 0.38 0.42 0.31 0.33 0.30 0.10
ERK1/2
1.47 1.97 1.63 1.54 1.49 1.48 1.34 1.34 1.14 1.50 1.46 1.63
P-ERK1/2
0.34 0.31 1.45 1.07 1.14 0.59 0.32 0.40 0.89 0.76 0.50 0.23
β-Actin

B

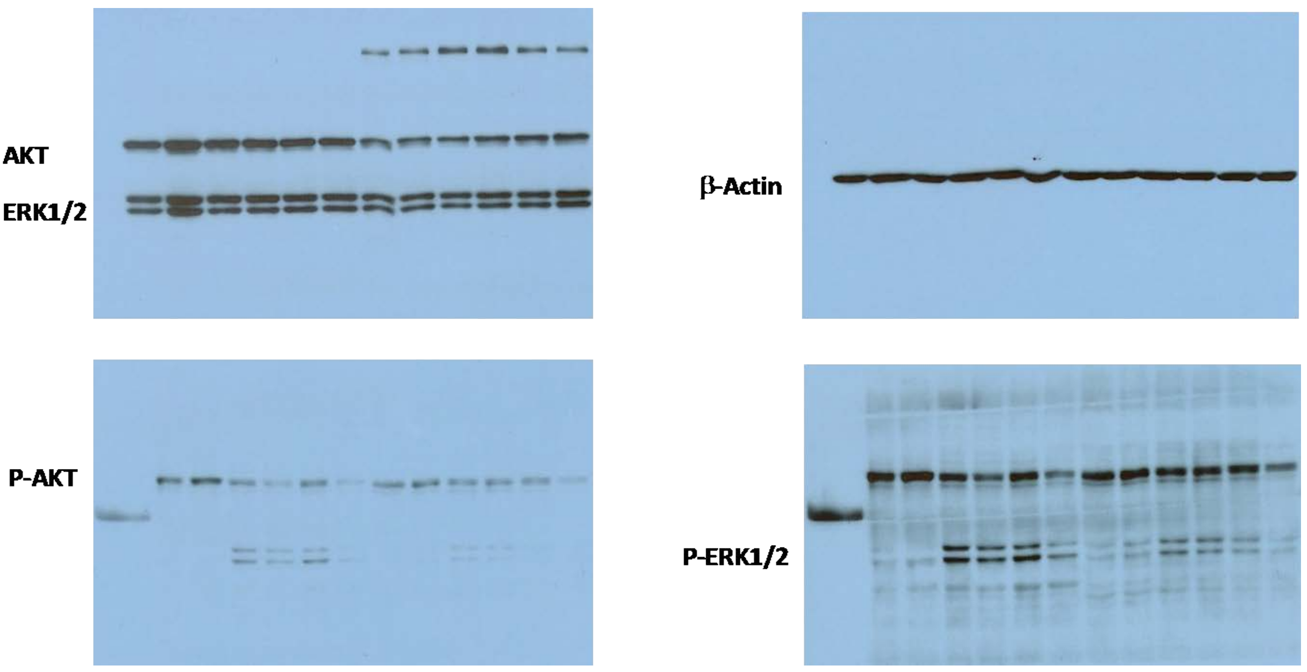
AKT
ERK1/2
β-Actin
P-AKT
P-ERK1/2

SUPPLEMENTAL TABLE S1

Date	Immunogen	Amount ug	Route Of Injection	Sera Id	Bleed Date
**Campaign 1**					
7/22/2016	LGals3 - Fc Fusion	50ug	SC (CpG+adjuvant)	Pre immune	7/22/2016
8/12/2016	LGals3 - Fc Fusion	50ug	SC (with adjuvant)	TB1	8/19/2016
9/2/2016	LGals3 - Fc Fusion	50ug	SC (with adjuvant)	TB2	9/9/2016
9/19/2016	LGals3 - Fc Fusion	50ug	SC (with adjuvant)	TB3	9/26/2016
10/10/2016	LGals3 - Fc Fusion	50ug (M2 M3)	SC (with adjuvant)	TB4	10/17/2016
10/18/2016	LGALS3 Protein	50 ug (M1, M4, M5)	SC (with adjuvant)	TB4	10/25/2016
10/31/2016	LGALS3 Protein	5ug	SC (with adjuvant)	TB5	11/7/2016
**Campaign 2**					
12/2/2016	Peptide - LGALS3 binding domain	50 ug	SC (with adjuvant)	TB6	12/9/2016
12/23/2016	Peptide - LGALS3 binding domain	50ug	SC (with adjuvant)	TB7	12/30/2016
1/13/2017	Peptide - LGALS3 binding domain	50 ug	SC (with adjuvant)	TB8	1/20/2017
4/7/2017	LGALS3 N-Terminus Peptide	50 ug	SC (with adjuvant)	TB9	4/14/2017
5/2/2017	LGALS3 N-Terminus Peptide	50 ug	SC (with adjuvant)	TB10	5/9/2017
5/23/2017	LGALS3 N-Terminus Peptide	50 ug	SC (with adjuvant)	TB11	5/30/2017
6/26/2017	LGALS3 N-Terminus Peptide	5 ug (M1)	IV - tail vein	Terminal	6/29/2017
6/26/2017	Peptide - LGALS3 binding domain	5 ug (M1)	IV - tail vein	Terminal	6/30/2017
**Campaign 3**					
12/16/2016	LGALS3 conserved region Peptide 1-KLH	20 ug	SC (with adjuvant)	Pre-immune	12/15/2016
12/30/2016	LGALS3 conserved region Peptide 1-KLH	50ug	SC (with adjuvant)	TB1	1/6/2017
1/20/2017	LGALS3 conserved region Peptide 1-KLH	50 ug	SC (with adjuvant)	TB2	1/27/2017
2/21/2017	LGALS3 conserved region Peptide 1-KLH	10 ug	IV - tail vein	Terminal	2/24/2017
4/7/2017	LGALS3 N-Terminus Peptide 9-KLH	50 ug	SC (with adjuvant)	TB3	4/14/2017
5/2/2017	LGALS3 N-Terminus Peptide 9-KLH	50 ug	SC (with adjuvant)	TB4	5/9/2017
5/23/2017	LGALS3 N-Terminus Peptide 9-KLH	50 ug	SC (with adjuvant)	TB5	5/30/2017
6/26/2017	LGALS3 N-Terminus Peptide 9-KLH	5 ug (M2)	IV - tail vein	Terminal	6/30/2017
